# Stereoscopic Visualization of Diffusion Tensor Imaging Data: A Comparative Survey of Visualization Techniques

**DOI:** 10.1155/2013/780916

**Published:** 2013-10-22

**Authors:** Osama Raslan, James Matthew Debnam, Leena Ketonen, Ashok J. Kumar, Dawid Schellingerhout, Jihong Wang

**Affiliations:** ^1^Department of Radiology, Section of Neuroradiology, MD Anderson Cancer Center, The University of Texas, 1400 Pressler Street, Unit 1482, Houston, TX 77030, USA; ^2^Deparment of Imaging Physics, MD Anderson Cancer Center, The University of Texas, 1400 Pressler Street, Unit 1482, Houston, TX 77030, USA

## Abstract

Diffusion tensor imaging (DTI) data has traditionally been displayed as a grayscale functional anisotropy map (GSFM) or color coded orientation map (CCOM). These methods use black and white or color with intensity values to map the complex multidimensional DTI data to a two-dimensional image. Alternative visualization techniques, such as *V*_max_ maps utilize enhanced graphical representation of the principal eigenvector by means of a headless arrow on regular nonstereoscopic (VM) or stereoscopic display (VMS). A survey of clinical utility of patients with intracranial neoplasms was carried out by 8 neuroradiologists using traditional and nontraditional methods of DTI display. Pairwise comparison studies of 5 intracranial neoplasms were performed with a structured questionnaire comparing GSFM, CCOM, VM, and VMS. Six of 8 neuroradiologists favored *V*_max_ maps over traditional methods of display (GSFM and CCOM). When comparing the stereoscopic (VMS) and the non-stereoscopic (VM) modes, 4 favored VMS, 2 favored VM, and 2 had no preference. In conclusion, processing and visualizing DTI data stereoscopically is technically feasible. An initial survey of users indicated that *V*_max_ based display methodology with or without stereoscopic visualization seems to be preferred over traditional methods to display DTI data.

## 1. Introduction

Diffusion tensor imaging (DTI) is a magnetic resonance (MR) imaging technique that enables the quantitative measurement of molecular diffusion in biologic tissues *in vivo* [1–7]. Diffusion signal changes are caused by the anisotropy (or directionality) of WM fibers; the fibers restrict water molecule movement across the axons while leaving movement along the axons relatively unrestricted. This results in unequal (or anisotropic) diffusivities along the axons. The ability to measure these very specific tissue characteristics *in vivo* is unique to DTI and has many applications in clinical neuroimaging including the delineation of tumor infiltration, assessing the integrity of neuronal fibers and neurosurgical planning [8–19].

Many techniques and schemes have been proposed for visualizing DTI data [20–28] such as using ellipsoids with their principal axes corresponding to the eigenvectors and using volumetric rendering or shading to present these ellipsoids' directional information [23]. These are rigorous approaches but are still subject to the limiting problems of any technique that visualizes 3D information in two dimensions (2D). Often the 3D shading of diffusion tensor ellipsoids [23] or superquadric glyphs [24] at all voxels does not give a global view of tensor data in an imaging plane or in a volume at the zoomed-out view. To get a true 3D perspective, one must use computer animation tools to rotate the image plane or image volume thereby obtaining multiple views of the 3D tensor data [20]. Research into new and intuitive methods of analyzing and representing DTI data continues [29].

DTI data needs to be displayed in an interpretable manner in order to support clinical decision making. This is a challenging proposition, because unlike other volumetric grayscale images, in which the value at each voxel can be represented by a single number (scalar), DTI image display needs to communicate multiple values reflecting directional and magnitude information at every voxel. In fact, the value at each voxel is a tensor, which requires the use of multiple parameters for its representation. A tensor is typically represented by a 3 × 3 matrix with a total of nine elements, of which six are independent. The tensor matrix can be diagonalized, yielding three eigenvalues and their corresponding eigenvectors. A full representation of DTI information would include all three eigenvalues and eigenvectors for every voxel.

**Figure 1:**
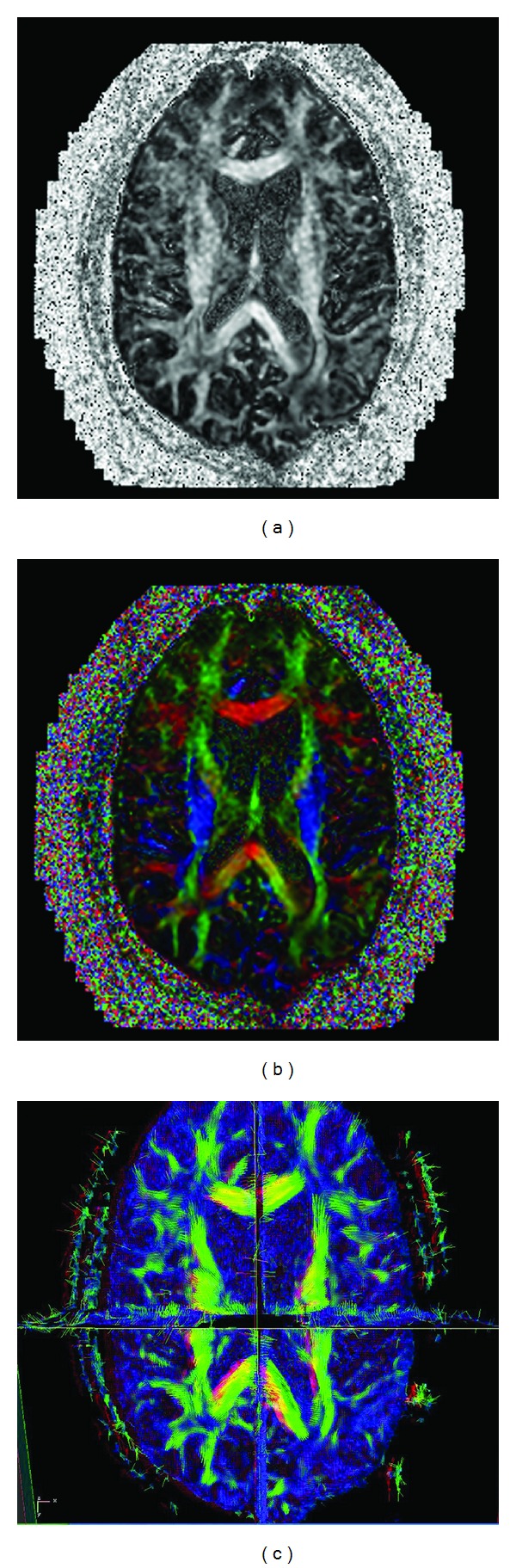
For comparison, the principal eigenvectors are presented in (grayscale (GSFA) map, color coded orientation view (CCOM), and headless arrow view (*V*_max⁡_)). (a) GSFA. The intensity represents the FA value while no directional information is given. (b) CCOM. In the color coded orientation view, which is commonly seen in today's DTI literature, blue color represents the craniocaudal (in-out of paper) direction, red represents the left-right direction, and green represents the anterior-posterior (up-down) direction. The mixed colors represent directions somewhere in between. The intensity of color represents the FA value. (c) *V*_max⁡_ map. In the stereoscopic view, eigenvectors' directional information is presented intuitively as headless arrows in 3D stereoscopically and colors represent the FA values.

In order to simplify this tensor dataset for display on a 2D grayscale image, grayscale fractional anisotropy (GSFA) maps are often constructed (Figure 1(a)). GSFA maps are simply plots of the fractional anisotropy (FA) values at those voxels with a large difference between the principal and other eigenvalues as bright values and those with little differences as dark values:

(1) $$\begin{array}{c} FA=\sqrt{\frac{1}{2}}\frac{\sqrt{{\left(\lambda_{1}-\lambda_{2}\right)}^{2}+{\left(\lambda_{1}-\lambda_{3}\right)}^{2}+{\left(\lambda_{2}-\lambda_{3}\right)}^{2}}}{\sqrt{\left(\lambda_{1}^{2}+\lambda_{2}^{2}+\lambda_{3}^{2}\right)}}, \end{array}$$

where *λ*_1_, *λ*_2_, and *λ*_3_ are three eigenvalues at each voxel.

Alternatively, color can be used to indicate direction and brightness to indicate the magnitude of the directional component, and color-coded orientation maps (CCOM) are displayed (Figure 1(b)).

Sometimes displaying all tensor information at every voxel at once may be cumbersome and even counterproductive for clinical diagnosis. Therefore, many have proposed and implemented visualization schemes that display only a portion of the tensor data [20–29]. For instance, in DTI-related publications, it is common to see fractional anisotropy (FA) maps, which are grayscale images of anisotropy indices that are composites of all three eigenvalues. As the principal eigenvector (*V*_max⁡_) represents the direction of fiber tracts, some DTI visualization schemes choose to present only the directional information of *V*_max⁡_ and another parameter that indicates anisotropy. One popular DTI representation scheme uses three colors to represent the directional information of *V*_max⁡_ in orthogonal planes [27].

Although color has been used extensively in medical imaging, sometimes as an indication of directional information, that is, color Doppler ultrasonography, many human observers are not sensitive to the subtle changes in color hue that represent directional information for DTI tensors. Therefore, the current, nontraditional technique of using colors to encode DTI eigenvector direction may not be intuitive, especially for eigenvectors with directions (CCOM) not parallel to any of the three orthogonal Cartesian coordinate axes, even with the use of a reference color circle [27].

**Figure 2:**
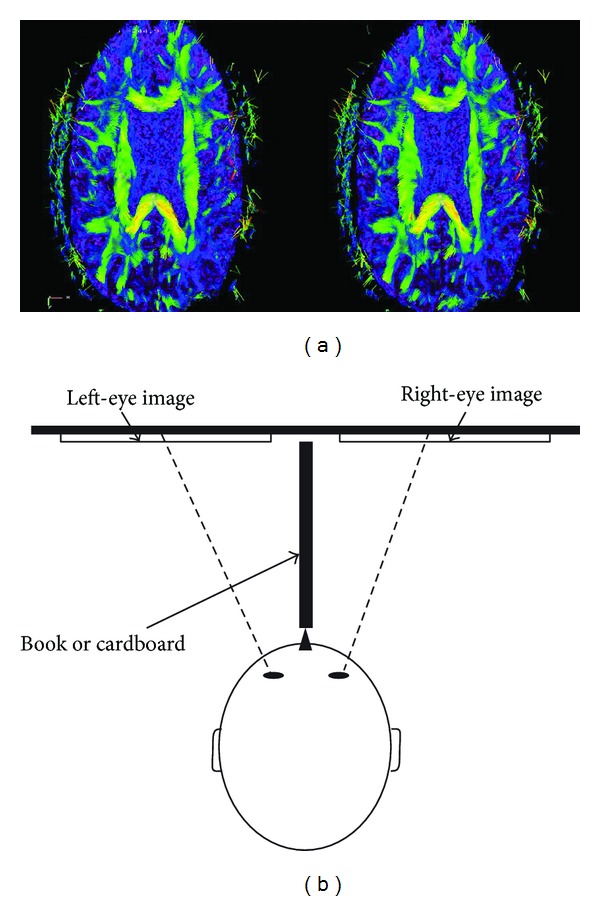
3D visualization tool. Stereoscopic left- and right-eye-image generated by the visualization tool; one can achieve the stereo effect by focusing beyond the image plane or with the help of a stereoscope, or by using a piece of cardboard put between the two images (as shown schematically in (b) the top-down view) such that each eye is able to see only one of the two images. Once the stereo effect is achieved, one can clearly appreciate the 3D effect of the headless arrows pointing out of the paper plane at various angles.

**Figure 3:**
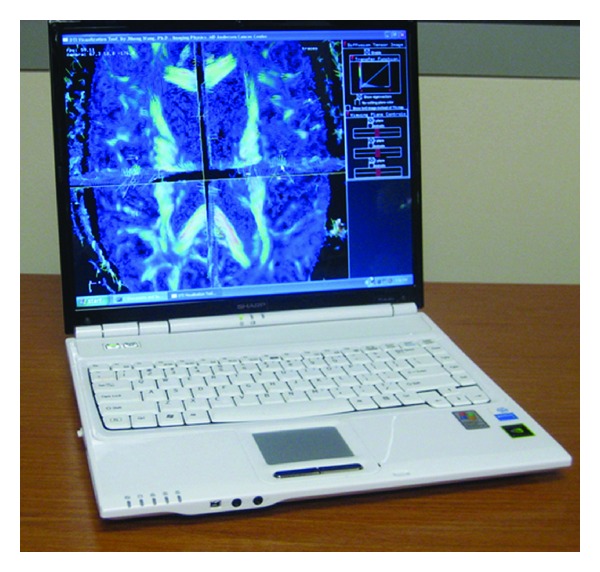
Photograph of the stereoscopic DTI processing and visualization tool. The laptop is equipped with a glasses-free LCD monitor. The laptop computer can operate in stereo or nonstereoscopic mode: the user can switch between modes by pushing a button. In stereoscopic mode, the LCD operates in 512 × 768 matrix size, and while in nonstereoscopic mode the LCD operates at 1024 × 768.

Alternatively, stereoscopic vision can also be employed; this adds another dimension to 2D grayscale images. For DTI, a natural and true 3D view of *V*_max⁡_, (Figure 1(c)) as well as the WM fiber tracts can be easily achieved using stereoscopic vision principles. Creating a stereoscopic image pair (i.e., the left- and right-eye views) and presenting the images to the respective eyes enables a human viewer with normal stereo vision to get a 3D perception of the spatial and directional information of diffusion tensors' eigenvectors and WM fiber tracts by reconstructing the left- and right-eye retinal images in his visual system (Figure 2). In theory, stereoscopic perceptions (VMS) can be generated by calculating the projected left- and right-eye-view images of the *V*_max⁡_ or WM fiber tracts and displaying these images with a stereoscopic image displaying device. Alternatively, we can put the pair of images side by side on paper (Figure 2) or on a computer monitor (Figure 3) and use a simple device (described below) that allows the left eye to see only the left-eye-view image and the right eye to see only the right-eye-view image. 

The purpose of this study was to assess the clinical utility of *V*_max⁡_ maps, both nonstereoscopic (VM) and stereoscopic (VMS), and to compare *V*_max⁡_ maps to traditional DTI display method (GSFA and CCOM).

## 2. Materials and Methods

Eight neuroradiologists participated in the study and evaluated the DTI of 5 patients (5 females, ages 4–62 years, mean 43 years, and median 47 years) with the following intracranial pathology: metastatic breast carcinoma, brainstem glioma, glioblastoma, anaplastic astrocytoma, and lymphoma. The patients were imaged with MRI scanners (GE Healthcare, Milwaukee, WI) using single-shot EPI pulse sequences to obtain DTI data (*b* value = 1200, number of diffusion gradient directions = 27, FOV = 22 cm, slice thickness of 3.5 mm with no gap, and matrix size 128 × 128). The same DTI image data set was generated for each patient with all four of the visualization techniques to be compared.

### 2.1. Conventional/Traditional DTI Processing Tool

A commercial DTI data processing software package (AW version 4.4, GE Healthcare, Milwaukee, WI) was used in the generation of the grayscale functional anisotropy maps (GSFM) and coded orientation maps (CCOM) images on the selected patients (Figures 1(a) and 1(b)). 

### 2.2. Stereoscopic DTI Processing

A DTI processing and stereoscopic visualization software was developed in-house with C++ (Microsoft Visual Studio, Microsoft Corp., Redmond, WA) using the OpenGL library for 3D rendering. From the diffusion weighted images, this software calculates the eigenvalues and eigenvectors as well as the ADC values first. Then it creates a headless arrow to represent the dominant eigenvector at each voxel, with the length of the arrow (as well as the color temperature) corresponding to the magnitude of the eigenvalue (VM). For stereoscopic view, the visualization program generates the two stereoscopic image pairs of these arrows at each voxel for all the image slices. Dominant principal eigenvector stereoscopic (VMS) images were generated by the stereoscopic visualization tool.

### 2.3. Visualization Tool

All images were transferred to a stereoscopically enabled laptop computer (PC-AL3DU laptop, Sharp Electronics Corp., Mahwah, NJ) (Figure 3). This device enables stereoscopic vision by a specially designed monitor. More specifically, stereoscopic viewing was achieved using Sharp's StereoGL and its glasses-free stereoscopic LCD monitor. For this stereoscopic monitor, a stereoscopic view was created by having the left- and right-eye views interlaced as the even and odd vertical lines on the monitor. At a certain viewing distance, the right-eye view is visible only to the right eye and the left-eye view is visible only to the left eye. This generates a 3D perception for human observers with normal stereoscopic vision without the use of polarizing glasses, and the visualization could be switched between stereoscopic (VMS) and nonstereoscopic (VM) mode with a control key button.

This software tool has two DTI data visualization functions: the eigenvector view function and the fiber-tracking (or tractography) function. In the eigenvector view function, the 3D vector fields of the principal eigenvectors (*V*_max⁡_) in the three orthogonal planes of the image volume are displayed stereoscopically. The eigenvectors are displayed as headless arrows and superimposed over the image of the FA values. The colors of the arrows correspond to the tensors' anisotropy indices (i.e., the FA values). Any of the three orthogonal planes can be turned on/off and looped through the image volume along with the eigenvectors in those planes. The tool also allows the user to zoom in on any point within the image volume for a detailed view of the eigenvectors and to rotate the image volume to view it from any angle. 

**Figure 4:**
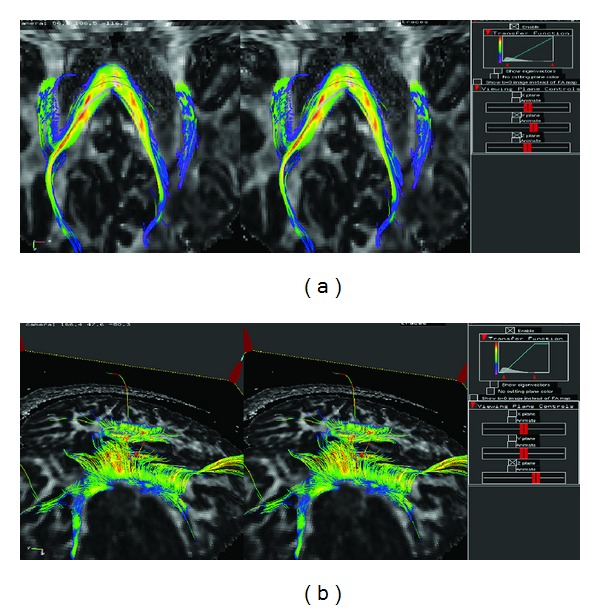
(a) DTI, fiber-tracking function. The fiber-tracking function of the DTI visualization tool is shown stereoscopically in the left-right eye stereo views. Notice the clear spatial separation of all the overlapping tracts once stereoscopic effect is achieved. (b) DTI, stereoscopic view. Color is used to depict other DTI parameters (such as the FA values in figures above) along the tracts.

In the fiber-tracking function (Figure 4(a)), this tool performs WM fiber tracking using a simple line-propagation algorithm that follows the paths of *V*_max⁡_ through the 3D tensor imaging volume. It assigns a color to each point along the fiber tracts corresponding to the local properties (e.g., FA value), using a typical rainbow color scale, and displays the tracts stereoscopically in 3D (Figure 4(b)). 

### 2.4. Clinical Study

The data sets for all 5 patients were presented to the neuroradiologists on the same computer upon which the stereoscopic visualization tool resides. This minimized any potential differences of preference due to computer monitor characteristics such as brightness and monitor sizes, luminance. Pairwise comparison utilization studies were performed by utilizing a structured questionnaire to assess the following visualization techniques: (a) GSFM, (b) CCOM, and (c) *V*_max⁡_ maps utilizing enhanced graphical representation of the principal eigenvector by means of a headless arrow without (VM) and with (VMS) stereoscopic display.

The 8 neuroradiologists were provided with a T1 postgadolinium image showing each of the five lesions, as well as the patient's demographics, the pathologic diagnosis of the lesion, and the lesion location (see Table 1). Paired sets of different DTI images for the five lesions were also provided, and arranged for direct comparison as follows: (study group 1: GSFM versus CCOM; study group 2: VM versus CCOM; and study group 3: VM versus VMS. 

### 2.5. Survey Questionnaire (Questions  1–3)

For each of the three tests of comparison images (study groups 1–3), the reviewers were asked the following three questions.

 Question 1: For the provided pair of images, which of the 2 is the most informative?
 Question 2: Rate the strength of your preference. [Equivalent (0), weak (1), moderate (2), strong (3)].
 Question 3: Explain the subjective reason for your choice.

### 2.6. Survey Questionnaire (Questions  4-5)

 Question 4: The neuroradiologists were asked to determine if the adjacent white matter tracts in cases 1 and 3 or middle cerebellar peduncles in case 2 were either (a) involved by the lesion, (b) displaced by the lesion, (c) not affected by the lesion, or (d) if the reviewers were unsure about tract involvement. For the other two cases, (cases 4 and 5), the neuroradiologists were asked which part of the corpus callosal fibers remained intact?
 Question 5: The reviewers were then asked about the confidence of their answer. [very unsure (1), unsure (2), undecided (3), confident (4), or very confident (5)].

The study questionnaire is summarized as follows.

 Question 1: For the current pair of images which is the most informative?
  (a) Image 1
  (b) Image 2
  (c) No difference.
 Question 2: Mark the box that best describes the strength of your preference. [(Equivalent (0), weak (1), moderate (2), strong (3)].
 Question 3: Can you explain why you made this choice?
 Question 4: Are the corticospinal tracts (cases 1 and 3) or middle cerebellar peduncles (case 2)?
  (a) Involved by the lesion
  (b) Displaced by the lesion
  (c) Not affected by the lesion
  (d) Not sure of the answer.
 Questions 4 (case 4): Which part of the Corpus Callosum fibers (CC) remains intact?
  (a) Rostrum
  (b) Genu
  (c) Body
  (d) Splenium
  (e) None
  (f) Not sure of the answer.
 Questions 4 (case 5): Which part of the Corpus Callosum fibers (CC) is affected?
  (a) Rostrum
  (b) Genu
  (c) Body
  (d) Splenium
  (e) None
  (f) Not sure of the answer.
 Questions 5: How confident are you of that answer? [(Very unsure (1), unsure (2), undecided (3), confident (4) very confident (5)].

## 3. Results

### 3.1. Questions  1-2

*Study Group 1—GSFM versus CCOM.* The CCOM was preferred in 70%, (28/40) (40 selections = 8 readers × 5 cases). The ranking for the strength of preference of CCOM (0–3) ranged between 2.3–2.8 (mean 2.5). The GFSA map and “no preference” between the two choices were selected in 15% of the cases each.

*Study Group 2—VM versus CCOM.* When comparing the VM nonstereoscopic to the CCOM maps, the VM nonstereoscopic was preferred in 80%, (32/40). The ranking for preference of VM nonstereoscopic (0–3) ranged between 2.2–2.7 (mean 2.5). The CCOM and “no preference” between the two choices were each selected in 10% of the cases. 

*Study Group 3—VM versus VMS*. When comparing the VM nonstereoscopic to the VM stereoscopic maps, the VMS stereoscopic was preferred in 45% (18/40) and the VM nonstereoscopic in 35% (14/40). There was no preference in 20% (8/40). The ranking for preference of VM stereoscopic (0–3) ranged between 1.5 and 3.0 (mean 2.1). These results for questions 1 and 2 are further summarized in Table 2.

### 3.2. Question  3 (Study Groups 1–3)

Subjective reason for preference of CCOM, VM nonstereoscopic and VM stereoscopic, respectively, included more information provided, better delineation of white matter tracts, and easier to determine loss of anisotropy. 

### 3.3. Questions  4-5

**Figure 5:**
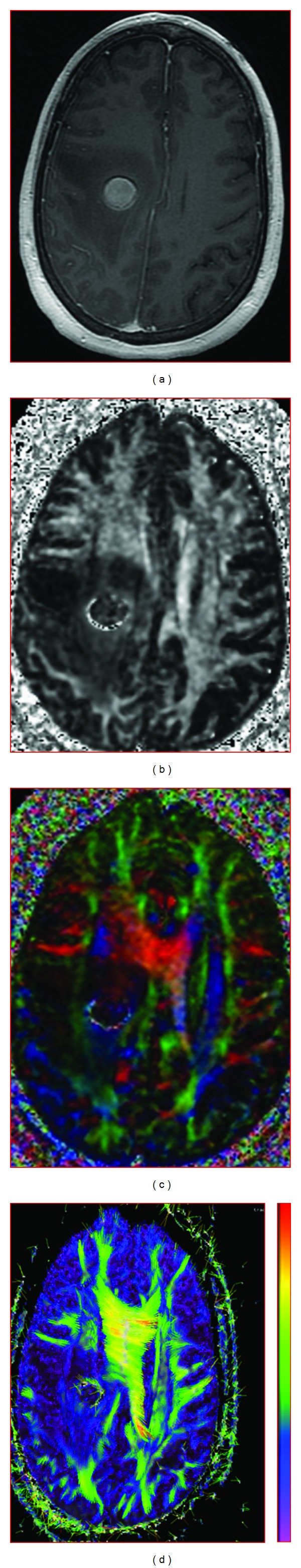
A 43-year-old female with breast cancer metastasis to the posterior right frontal lobe. (Case 1): (a) axial T1 postgadolinium; (b) grayscale FA (GSFA) map; (c) color coded orientation map (CCOM); and (d) stereoscopic view. There was an increase from 5 of 8 to 8 of 8 correct responses regarding white matter tract involvement between the GSFA map and VM nonstereoscopic and stereoscopic maps.

**Figure 6:**
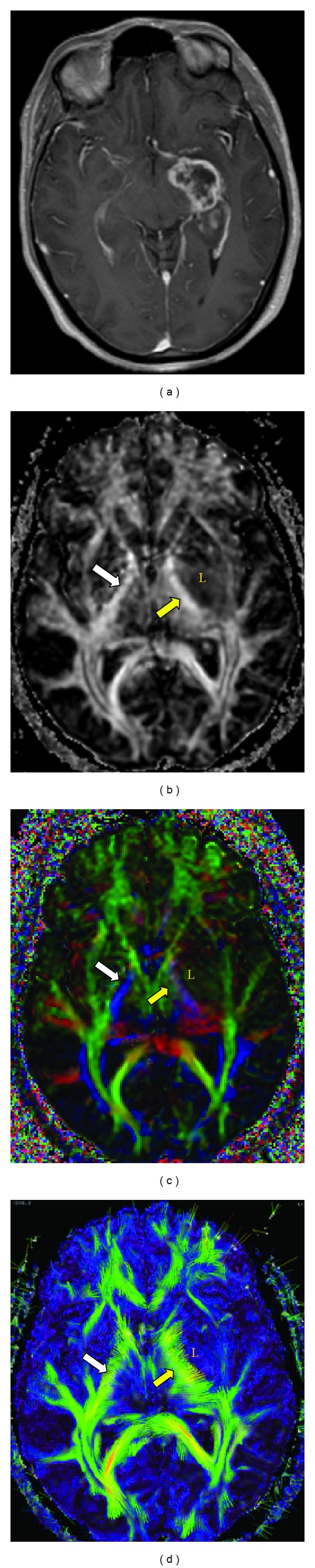
A 47-year-old female with left temporal glioblastoma multiform (L). (Case 3): (a) axial T1 postgadolinium; (b) grayscale FA (GSFA) map; (c) color coded orientation map (CCOM); and (d) nonstereoscopic *V*_max⁡_ view. The question is the left corticospinal tract (CST) (yellow arrow) affected by the lesion or not? The CCOM suggested that the CST was affected by the lesion evidenced by change in its blue color (i.e., fibers have changed their direction), as well as the intensity of the blue color (i.e., fibers have changed their FA value); so conclusion was the CST is infiltrated by the lesion. The *V*_max⁡_ images suggest that the ipsilateral CST is almost normal, apart from being slightly displaced, and having a higher FA values compared to opposite side (more yellow areas); so the conclusion was that the fibers are only displaced. The higher FA value could reflect the fibers becoming more compact as a result of the mass effect. To resolve this discrepancy, we resorted to the GSFA images as well as the clinical data. The GSFA showed that both CST have almost the same intensity (i.e., FA values) (white arrow = normal CST), so our conclusion was that the fibers are most likely not infiltrated. A through clinical exam revealed that there is no motor affection.

In case 1 (Figure 5), when the neuroradiologists compared the GSFA maps to the CCOM, only 5 of 8 reviewers correctly identified that white matter tracts were invaded by the lesion. The number of reviewers who correctly identified involvement of the tracts rose to 7 of 8 with the CCOM maps and to 8 of 8 with the VM and VMS maps. In case 3 (Figure 6), 3 of 8 reviewers correctly identified that there was displacement of the white matter tracts when reviewing the GSFA maps. This increased to 4 of 8 with the CCOM, VM, and VMS maps. 

In the other 3 test questions (cases 2, 4, and 5), there was no difference in detection of white matter tract involvement between the 4 sets of DTI display maps. The results for the test questions and confidence ranking of the reviewers are summarized in the accompanying Table 3.

## 4. Discussion

Despite the relatively small sample size, our preliminary results demonstrate that the traditional color-coded DTI display (CCOM) seems preferred over the grayscale display (GSFA), and that the nontraditional *V*_max⁡_ maps (VM and VMS) seem preferred over the traditional color-coded display (CCOM). The subjective reasons for the preference of color-coded display and the *V*_max⁡_ maps includes better delineation of white matter tracts and ease in determining loss of anisotropy. In 2 of 5 test cases (patients 1 and 3), the color-coded display and *V*_max⁡_ maps provided better demonstration of white matter tract displacement or involvement. All the neuroradiologists who used this stereoscopic DTI visualization tool were able to achieve the stereoscopic effect with ease, and all stated that stereoscopic display of DTI eigenvectors was more intuitive than the color-encoded method (CCOM).

Traditionally, medical images ranging from X-ray films to computed tomography (CT) and MR images are presented in grayscale. Historically, the reason stemmed from the use of black and white film as the medium for X-ray images, but displaying medical images in grayscale was and still is an intuitive way to visualize scalar images (i.e., images in which each pixel can be represented by a single number) such as X-ray and later CT and MR images. Although there were past attempts to use color or pseudocolor in place of grayscale, they did not take hold in displays of scalar images. The intensity in X-ray and CT images often corresponds to some underlying physiological process, such as tissue density. When intensity is considered with spatial distribution (the morphology), the underlying disease process can often be diagnosed. 

With the addition of functional and dynamic imaging in recent decades, imaging data became multidimensional and alternative ways to visualize these data had to be implemented. Color, and sometimes the intensity of color, has been successfully employed in the visualization of some multidimensional data, such as in color Doppler ultrasonography. In color Doppler images, a binary color visualization scheme (red and blue) is employed to represent the direction of blood flow in arteries and veins, and the intensity represents the flow speed. Color adds a degree of freedom (or a dimension) in the data representation. Similarly, stereovision can potentially add a degree of freedom in the representation of multidimensional image data. 

The current popular visualization tool CCOM uses one dimension, which is the color to represent two variable indices, the direction and the FA value of the axons. Although this might seem to be a more intuitive way to interpret the DTI, great caution must be taken when analyzing the data using this tool that is, when you have a change in the color, should we interpret this as change in the direction of the fibers, in the FA value of the fiber or both? Should we interpret this as infiltration of the fibers by the lesions, or just mere displacement? Further study may provide answers to these questions. Other topics for future investigation may include determining if this technique is applicable in the operating room during the neurosurgical procedures, and if any benefit is added by reviewing diffusion tensor glyphs to the headless arrow. 

An obvious advantage of utilizing a stereoscopic view in DTI is that even with a single pair of static images (Figure 3) users can perceive the orientation of the eigenvectors plus the relative spatio-directional information of the WM tracts in the tractography mode. This enables an intuitive visual assessment of FA values along the WM tracts. Since FA values are believed to correlate with some physiological properties of WM tracts, one may use this tool to assess the integrity of WM tracts near or inside a tumor, leading to potential applications in treatment planning and assessment of treatment efficacy in patients with brain tumors. We believe that with the colors of the WM tracts indicating FA values and the stereoscopic views providing spatial information about the tumor volume and the tracts, a user will get a more intuitive visual assessment of the changes (or lack of changes) in the FA values along the WM tracts as they pass near or through a tumor. 

## 5. Conclusion

Processing and visualizing DTI data stereoscopically is feasible, and an initial survey of users indicated that stereoscopic DTI visualization is more intuitive than the color coded orientation maps to humans with normal stereoscopic vision. The tool enables the use of color to indicate the FA values along WM fiber tracts, providing an intuitive technique to visually assess the integrity of the tracts. 

## Figures and Tables

**Table 1:** Patient demographics.

Patient no.	Age/sex	Lesion	Location
1	43/F	Breast cancer metastasis	Right frontal
2	4/F	Glioma	Brainstem
3	42/F	Glioblastoma	Left temporal lobe
4	60/F	Astrocytoma	Right frontoparietal
5	62/F	Lymphoma	Corpus callosum

**Table 2:** Study results—questions 1 and 2.

Case	Study group 1		Study group 2		Study group 3	
	GSFA versus CCOM		CCOM versus VM		VM versus VMS	
	Prefer	Rank (0–3)	Prefer	Rank (0–3)	Prefer	Rank (0–3)
1	CCOM 8/8	2.38	VM 6/8	2.67	VMS 4/8	1.75
2	CCOM 5/8	2.8	VM 5/8	2.2	VMS 4/8	1.5
3	CCOM 5/8	2.6	VM 7/8	2.57	VMS 3/8	3
4	CCOM 7/8	2.29	VM 6/8	2.5	VMS 3/8	2
5	CCOM 3/8	2.33	VM 8/8	2.38	VMS 4/8	2.25

**Table 3:** Study results—questions 4 and 5.

Case	Test 1	Test 1	Test 2	Test 2	Test 3	Test 3
	Tract involvement		Tract involvement		Tract involvement	
	Correct-Incorrect-Unsure	Confidence (1–5)	Correct-Incorrect-Unsure	Confidence (1–5)	Correct-Incorrect-Unsure	Confidence (1–5)
1	5-1-2	4	7-1-0	3.57	8-0-0	3.38
2	7-1-0	3.43	7-1-0	3.29	7-1-0	3.57
3	3-5-0	3.67	4-4-0	3	4-4-0	3.25
4	8-0-0	3.75	8-0-0	3.13	8-0-0	3.25
5	7-0-1	3.71	7-0-1	3.57	7-1-0	3.43
